# Oncofetal antigens as emerging targets in chimeric antigen receptor (CAR)-T cell-based immunotherapies: opportunities and challenges

**DOI:** 10.3389/fcell.2026.1910208

**Published:** 2026-09-04

**Authors:** Sam Sebastian Smith, Astero Klampatsa

**Affiliations:** 1 Royal Marsden Hospital, London, United Kingdom; 2 The Institute of Cancer Research, London, United Kingdom

**Keywords:** CAR, CAR-T therapy, CAR (chimeric antigen receptor), oncofetal, oncofetal antigen

## Abstract

Oncofetal antigens are emerging targets for chimeric antigen receptor (CAR)-based immunotherapies due to their developmentally restricted expression and frequent re-emergence in malignant cells. These antigens are tightly linked to cellular plasticity, stem-like programs, immune evasion, and resistance to cytotoxic therapies, making them biologically compelling targets. Multiple oncofetal antigens have entered early-phase clinical trials as CAR-T cell targets with promising results in several cancers including neuroblastoma and diffuse midline gliomas. However, antitumor efficacy in most solid malignancies has thus far been modest. This reflects shared obstacles, including low-level expression in normal tissues, intratumoral antigen heterogeneity, immune suppression within the tumor microenvironment, CAR-T cell exhaustion, and limited persistence. In this Mini-Review, we discuss the biological foundations of oncofetal antigen expression, synthesize clinical and pre-clinical experience targeting these antigens with CAR-T cell therapies, and highlight emerging engineering and combination strategies designed to overcome current limitations. We propose that integrating principles from developmental biology, tumor evolution, and immuno-engineering will be essential to unlocking the therapeutic potential of oncofetal antigen-directed CAR-T therapies in solid tumors.

## Introduction

Chimeric Antigen Receptor (CAR)-T cell therapy is a cell-based immunotherapy where T cells are genetically engineered to recognize and eliminate cells expressing a specific cell surface target. CAR-T cell therapy has transformed the treatment landscape in several hematologic malignancies with (currently) seven food and drug administration (FDA)-approved products delivering high response rates and durable remissions in otherwise refractory disease (Zi et al., 2026). However, translating this success to solid tumors has proven difficult. Unlike hematologic cancers, solid tumors present obstacles including poor immune infiltration, immunosuppressive tumor microenvironments (TMEs), tumor antigen heterogeneity and tumor cell escape mechanisms (Escobar et al., 2025). Another critical distinction between hematologic and solid tumors is the availability of lineage-restricted targets. Antigens such as CD19 and B-cell maturation antigen (BCMA) are largely confined to B-cell lineages and cells expressing these antigens can be eliminated with manageable consequences (Kochenderfer et al., 2010). In solid tumors, most candidate antigens are shared with essential normal tissues, resulting in narrow therapeutic windows and unacceptable toxicity when targeted aggressively (Escobar et al., 2025).

Oncofetal antigens offer a potential solution to this challenge. These proteins are expressed during embryonic/fetal development, epigenetically silenced in most differentiated adult tissues, and re-expressed in cancer. Importantly, expression of oncofetal antigens is commonly enriched in poorly differentiated and treatment-resistant cancers - the context in which new therapies are most needed (Moorman et al., 2025). Their developmental restriction suggests tumor specificity, while their association with aggressive tumor behavior implies biological relevance to malignant progression (Nguyen et al., 2026). Despite their favorable profile oncofetal antigen-directed CAR-therapies have produced limited clinical benefit to date. Understanding why requires consideration not only of antigen expression patterns, but also of immune tolerance, tumor cell plasticity, selective pressure, and CAR-T cell biology. In this Mini Review we will synthesize the current knowledgebase and emerging advances in CAR-T targeting of oncofetal antigens for solid tumors.

## Oncofetal antigen biology

### Oncofetal reprogramming and cancer plasticity

Oncofetal antigens are typically expressed by cancer cells that have undergone oncofetal reprogramming (Nguyen et al., 2026), a process conferring a phenotype that is beneficial to the cancer and includes resistance to toxic substances, immune modulation, rapid proliferation and angiogenesis (Cao et al., 2023). Oncofetal reprogramming is increasingly recognized as a dynamic and reversible cell state rather than a fixed differentiation endpoint (Moorman et al., 2025). Within a single tumor, subsets of cells may transiently adopt fetal-like programs in response to hypoxia, chemotherapy, nutrient deprivation, or immune pressure. This plasticity contributes to intratumoral heterogeneity and facilitates tumor evolution. Importantly, oncofetal cell states are frequently enriched following treatment, suggesting that oncofetal antigen expression may mark residual disease populations responsible for relapse (Moorman et al., 2025). This association strengthens the rationale for targeting oncofetal antigens therapeutically, but also introduces complexity, as selective pressure against these states may drive tumor adaptation.

Individual oncofetal antigens demonstrate dynamic expression changes in response to varying stimuli, including cancer treatments. For example, carcinoembryonic antigen (CEA) expression is increased in response to both chemotherapy and radiotherapy (Prete et al., 2008; Mueller et al., 2020). The plasticity of oncofetal antigen expression likely reflects epigenetic modulation, with oncofetal antigens such as claudin-6 (CLDN6) downregulated by promoter methylation and upregulated by treatment with a methyltransferase inhibitor which causes demethylation (Liu et al., 2016). Antigen density is key to CAR-T activity and the timing and utility of oncofetal antigen targeting CAR-T therapy may be dramatically affected by prior or concurrent therapies (Majzner et al., 2020).

### Immune tolerance and implications for CAR design

From an immunological standpoint, oncofetal antigens are self-proteins expressed during thymic development. As a result, high-affinity conventional T-cell receptors (TCRs) recognizing these antigens are deleted or tolerized through central and peripheral tolerance mechanisms limiting endogenous T-cell responses against them (Xing and Hogquist, 2012). CARs bypass these barriers by coupling antibody-derived antigen recognition domains to potent T-cell signaling modules, enabling major histocompatibility complex (MHC)-independent recognition of self-derived proteins. While this allows effective targeting of oncofetal antigens, it removes the safety constraints imposed by tolerance, increasing the risk of on-target off-tumor toxicity when antigen expression is not strictly tumor-restricted (Flugel et al., 2023). Therefore, the suitability of an oncofetal antigen for CAR-targeting depends not only on differential expression between tumor and normal tissues, but also on expression density, tissue distribution, molecular homology, and the ability to engineer CARs with appropriate sensitivity and specificity.

## Clinical experience with oncofetal antigen-targeted CAR-T cells

Oncofetal antigens need to be expressed on the cell surface for CAR-targeting. Several oncofetal antigens have advanced to clinical trials, and we will discuss these here (Table 1).

**TABLE 1:** Published clinical trials of CAR-T cells targeting oncofetal antigens.

Target	Phase	Population	CAR intracellular signaling domains	Engineering/combination	Local or systemic administration	Objective response rate (all patients/patients receiving optimal or maximum tolerated dose)	Disease control rate (all patients/patients receiving optimal or maximum tolerated dose)	Optimal dose or not found (% receiving optimal or maximum dose where not found)	Hematological toxicity (all patients/patients receiving optimal or maximum tolerated dose)	Non-hematological toxicity (all patients/patients receiving optimal or maximum tolerated dose)	References
CEA	1	CEA + liver metastases n = 8 (6 colon, 1 gastric, 1 ampullary)	CD28 + CD3ζ	IL-2	hepatic artery infusion	0% (0%)^†^	12.5% (0%)	3 infusions of 10X10^9 cells (37.5%)	0% (0%) G4 12.5% (33%) G3	43% (67%) G3 abdominal pain 43% (67%) G3 emesis	(Katz et al., 2015)
CEA	1	CEA + metastatic CRC n = 10	CD28 + CD3ζ	-	IV	0% (0%)	70% (100%)	not found (20%)	90% (100%) G3/4	10% (0%) duodenal perforation	(Zhang et al., 2017)
CEA	1	CEACAM + tumors n = 14 (6 CRC, 3 gastric, 1 GOJ, 2 esophageal, 1 pancreas, 1 pseudomyxoma peritonei)	CD3ζ	IL-2	IV	0% (0%)	21.4% (57.1%)	likely 5X10^10 T cells (50%)	85.7% (85.7%) G3/4*	28.5% (57.1%) G3/4 respiratory toxicity*	(Thistlethwaite et al., 2017)
CEA	1	CEA + CRC with liver metastases n = 8	CD28 + CD3ζ	SIRT IL-2	hepatic artery infusion	0% (?)^††^	16.7% (?)	3 infusions of 10X10^9 cells (75%)	0% (0%) G4 33% (33%) G3	33% (33%) G3 colitis 50% (50%) G3 fever	(Katz et al., 2020)
CEA	1	CEA + metastatic solid tumor n = 45 (36 CRC, 3 gastric, 2 appendiceal, 2 lung, 1 pancreatic, 1 cholangiocarcinoma)	4-IBB + CD3ζ	hypoxia responsive CAR-T	IV (60.5%), IP (39.5%)	IV 8% (0%), IP 23.5% (18.2%)	IV 68% (66.7%), IP 82.4% (81.8%)	3X10^6 CAR + per kg IP (64.7%), 5X10^6 CAR + per kg IV (36%)	100% (100%) G3/4 IV and IP	IV – 11% (22.2%) G3 diarrhea, 7.7% (11.1%) G3 oral/pharyngeal ulcer IP- 35% (27.3%) G3 diarrhea, 23.5% (18.2%) G3 oral/pharyngeal ulcer	(Gao et al., 2026)
ROR1	1	Cohort B n = 18: ROR1+ metastatic solid tumors (10 TNBC, 8 NSCLC)	4-IBB + CD3ζ	-	IV	5.60% (?)	55.60% (?)	likely 3.3X10^6/kg cells (52.4%)	38% (?) G3 anemia 29% (?) G3 febrile neutropenia**	5% (?) G5 respiratory failure 66.7% (?) G3/4 pulmonary toxicity**	(Jaeger-Ruckstuhl et al., 2025)
TAG72	1	TAG72+ CRC with liver metastases n = 23	CD3ζ	IFNα	IV (60.9%), hepatic artery infusion (39.1%)	0% (?)	0% (?)	Not found (?)	?	10% G3 retinal occlusion 10% G3 chills*	(Hege et al., 2017)
GPC3	1	GPC3+ HCC (n = 13)	CD28 + CD3ζ	-	IV	15.4% (25%)	30.8% (25%)	2X10^9/kg CAR+ (38.5%)	92.3% (100%) G3/4	7.7% (20%) G5 CRS 7.7% (20%) G4 hyperbilirubinemia 7.7% (20%) G3 hyperbilirubinemia	(Shi et al., 2020)
GPC3	1	GPC3+ advanced HCC (n = 6)	4-IBB + CD3ζ	RUNX-3 co-expression	IV	16.7% (?)	50% (?)	250 X10^6 CAR+ (100%)	100% (100%) G3/4	50% (50%) G3/4 CRS 17% (17%) G3/4 anaphylactic shock 33% (33%) G3/4 pneumonia	(Fu et al., 2023)
GPC3	1	GPC3+ metastatic cancer n = 24 (12 not armored (5 HB, 6 HCC, 1 HM) 12 armored (6 HCC, 2 HM, 1 Wilms tumor, 1 pNET, 1 YST, 1 ER)	4-IBB + CD3ζ	IL-15 armored (50%)	IV	non-armored 0% (?), armored 33% (33%)	non-armored 50% (?), armored 66.7% (66.7%)	not found (50% non-armored, 100% armored)	Non-armored 100% (100%) G3/4, armored 91.7% (91.7%) G3/4	Non-armored – 16.7% (?)G4 hypokalemia Armored – 16.7% (16.7%) G4 CRS, 8.3% (8.3%) G4 hypoxia	(Steffin et al., 2025)
CLDN6	1	CLDN6+ metastatic cancers n = 22 (13 germ cell testicular, 4 epithelial ovarian, 1 endometrial, 1 serous fallopian tube, 1 DSRCT, 1 gastric cancer, 1 CUP)	4-IBB + CD3ζ	RNA vaccine	IV	24% (46.7%)^†††^	67% (80%)	not found (68.2%)	?	5% (?) G3/4 CRS 5% (13.3%) G4 HLH 32% (?) G3/4 lipase increase	(Mackensen et al., 2023)
GD2	1/2	relapsed, refractory or high-risk neuroblastoma n = 54 (35 treated within trial, 19 in hospital exemption setting)	CD25 + 4-IBB + CD3ζ	-	IV	66%***(?)^††††^	84.4%***(?)	10X10^6 CAR per kg (82.9% of trial patients)	72.2% (?) G3/4	7.4% (?) G3 ICANS, 25.9% (?) G3/4 elevated transaminases, 1.9% (?) brain hemorrhage, 1.9% (?) G3/4 cardiac blood clot	(Locatelli et al., 2025)
GD2	1	H3K27M mutant diffuse midline glioma (n = 11)	4-IBB + CD3ζ	-	IV and intracerebroventricular if benefit from IV	36.4% (0%)^†††††^	72.7% (66.7%)	1X10^6/kg CAR+ (27.2%)	100% (100%) G3/4	9.1% (?) G3 ICANS, 9.1% (333.%) G5 brain hemorrhage, 54.5% (?) G3/4 hydrocephalus, 72.7% (?) G3/4 TIAN	(Monje et al., 2025)
GD2	1	GD2 positive solid cancers (n = 12) (5 BRAF mutant melanoma, 2 BRAF WT melanoma, 4 colorectal cancer, 1 sarcoma)	OX40 + CD28 + CD3ζ	BRAF/MEKi in BRAF mutant melanoma patients	IV	0% (0%) without BRAF/MEKi, 100% (100%) with BRAF/MEKi	28.6% (33.3%) without BRAF/MEKi, 100% (0%) with BRAF/MEKi	not found (25%)	0% (0%) G3/4	0% (0%) G3/4	(Gargett et al., 2024)

* (limited safety data provided) ** side effects from 21 patients who received ROR1 CAR-T, cells across cohort B (n = 18) and cohort A of patients with chronic lymphocytic leukemia (n = 3). The protocol was consistent between cohort A and B apart from lymphodepletion (100% of cohort A received cyclophosphamide and fludarabine, whereas 61.1% of cohort B received this regimen and 38.9% received cyclophosphamide and oxaliplatin). *** (32 patients treated within trial with evidence of disease used for efficacy analyses) ^†^ One patient was alive with disease 102 weeks after treatment having had stable disease and subsequent microwave ablation of residual disease and systemic therapy. ^††^ One patient had a complete metabolic response and survived for 30.8 months following CAR-T, administration. ^†††^ One patient had a complete response ongoing after 10.5 months. ^††††^ Three patients who achieved maintained PR/SD, remained alive with no further treatments and without evidence of progression 4–5.5 years after CAR-T, administration. ^†††††^ One patient achieved a complete response ongoing after 30 months.

### Carcinoembryonic antigen (CEA)

CEA was the first oncofetal antigen to be identified and remains one of the most extensively evaluated. It is expressed in a wide range of epithelial malignancies, particularly colorectal adenocarcinoma, but is also detectable at low levels in normal gastrointestinal and respiratory epithelium. In a tissue microarray CEA was detectable in 31.5% of 13,725 tumors and 65 of 120 tumor types (Jansen et al., 2024). In fetal development and healthy adult tissues, CEA functions primarily in cell adhesion. In adult cancers, its over-expression promotes disease progression by enhancing tumor cell adhesion, inhibiting apoptosis, and facilitating immune evasion (Sun et al., 2026). Clinical trials of CEA-targeted CAR-T cells, administered both systemically and locoregionally, demonstrated limited antitumor responses (Katz et al., 2015; Zhang et al., 2017). A major challenge with targeting CEA is its high normal tissue expression; a trial of 1^st^ generation CEA targeting CAR-T cells was prematurely closed due to acute respiratory toxicity (Thistlethwaite et al., 2017).

### Claudin-6

Claudin-6 is a more recently identified oncofetal protein expressed in multiple fetal tissues but almost absent in children and adults. It is re-expressed in several pediatric and adult cancers including germ cell, ovarian and endometrial cancers (Seidmann et al., 2025; Reinhard et al., 2020). In health, the tight junction protein claudin-6 has roles in fetal epithelial differentiation (Sugimoto et al., 2013). In malignancy, claudin-6 promotes cancer cell survival, proliferation and invasion (Wu et al., 2025). A recent phase 1 trial of a CLDN6 targeting CAR-T (combined with an RNA vaccine) in adults with CLDN6+ tumors revealed manageable toxicity and evidence of efficacy with an unconfirmed objective response rate (ORR) of 33% (Mackensen et al., 2023). While this trial is ongoing (NCT04503278) a planned phase 2 trial in patients with germ cell tumors (NCT06940804) has been withdrawn before recruitment of any patients.

### Glypican-3 (GPC3)

GPC3 is a heparan sulfate proteoglycan which is expressed during fetal liver development and re-expressed in hepatocellular carcinoma (HCC) and select other malignancies (Zheng et al., 2022). There is however expression in multiple normal tissues including the lung (Zheng et al., 2022). GPC3 is thought to regulate cell proliferation and apoptosis during embryogenesis (Piao et al., 2025). In cancer, GPC3 regulates signaling pathways including Wnt, and Hippo/YAP to avoid apoptosis and promote the epithelial-mesenchymal transition (EMT) (Piao et al., 2025). Unfortunately, efficacy of GPC3 targeted CAR-T cells has been limited. GPC3 CAR-T cells tested in a phase 1 trial induced no antitumor responses but were well tolerated (Steffin et al., 2025).

### Receptor tyrosine kinase-like orphan receptor 1 (ROR1)

ROR1 is a type 1 receptor tyrosine kinase highly expressed in multiple tumors including lung and breast, with limited normal tissue distribution (Balakrishnan et al., 2017). In embryogenesis, ROR1 has important roles in neurological, cardio-respiratory and skeletal development (Borcherding et al., 2014). In solid tumors, ROR1 has several roles including regulating apoptosis and potentiating epidermal growth factor receptor (EGFR) signaling (Borcherding et al., 2014). A phase 1 trial of ROR1 CAR-T cells in triple-negative breast cancer and non-small-cell lung cancer (NSCLC) demonstrated manageable toxicity but minimal efficacy, with only one patient having a short-lived partial response (Jaeger-Ruckstuhl et al., 2025).

### Tumor associated glycoprotein (TAG)72

TAG72 was identified as an oncofetal antigen in 1986, with minimal normal tissue expression but overexpression in multiple carcinomas including breast, colon, and NSCLC (Thor et al., 1986). TAG72 is normally expressed in secretory phase endometrium and induces anti-inflammatory dendritic cells in early pregnancy (Laskarin et al., 2011). Its role in cancer is unclear but may relate to immune evasion. The first human trials of CAR-T cells in the 1990s targeted TAG72 and while there was no clear on-target off-tumor toxicity there were no radiological responses (Hege et al., 2017). Development of TAG72 as a CAR-target has been limited until the last decade, but there is now at least one ongoing clinical trial of CAR-T cells targeting TAG72 (NCT05225363).

### Ganglioside G2 (GD2)

GD2 is a glycosphingolipid with high expression on neuroectodermal cells in fetal development and lower expression in adult tissues, e.g., the CNS (Lammie et al., 1993). GD2 is commonly upregulated on tumor cells arising from the neuroectoderm (melanoma, neuroblastoma, small cell lung cancer) (Nazha et al., 2020). In embryogenesis, GD2 has roles in neuronal differentiation (Jin et al., 2010). In cancer, GD2 has roles in adhesion, angiogenesis, cell survival and the TME (Machy et al., 2023). GD2 CAR-T cells have been highly effective in children with high risk metastatic, relapsed or refractory neuroblastoma with 5-year OS of 68% (Locatelli et al., 2025). GD2 CAR-T cells also show promise in the treatment of diffuse midline gliomas, with one patient in a phase 1 trial demonstrating an ongoing complete response 30 months after treatment (Monje et al., 2025).

### Emerging oncofetal antigen targets

There are several other oncofetal antigens that have been tested as CAR-targets in pre-clinical settings, including those listed below.

### 5T4 (trophoblast glycoprotein)

5T4 is a glycoprotein found in embryonic cells and trophoblasts. It is highly expressed in many cancers including multiple soft tissue sarcomas (Groothuis et al., 2021) with low expression in several adult tissues (Southall et al., 1990). In fetal development, 5T4 is thought to be important in promoting cell migration (Zhao and Wang, 2007). In malignancy, 5T4 has roles in EMT, migration, and regulating Wnt signaling (Stern and Harrop, 2017). Second-generation 5T4 targeting CAR-T cells have been found to have activity against cancer cells *in vitro* and *in vivo* (Guo et al., 2020; Owens et al., 2018).

### Oncofetal tenascin-C (TNC)

TNC is a secreted extracellular matrix (ECM) protein that binds to cells via surface receptors and has membrane localization on immunohistochemistry (Wickman et al., 2024). Multiple isoforms of TNC exist in humans, and an isoform containing the extra domain C (EDC-TNC) is oncofetal, found in fetal development and multiple cancers including lung and brain with very low normal tissue expression (Silacci, 2006; Wickman et al., 2024). In health, TNC regulates proliferation, adhesion and maintains inflammation (Wang et al., 2025). In malignancy, TNC has roles including regulating the TME, EMT and promoting immune evasion (Wang et al., 2025). EDC-TNC targeted CAR-T cells showed specific killing of EDC-TNC positive tumor cells (Wickman et al., 2024).

### Extra-domain B of fibronectin (EDB-FN)

EDB-FN is an oncofetal splice variant. It has low expression in adult tissues (limited to the gastrointestinal tract and ovaries), but is highly expressed in many cancers including NSCLC, breast, ovarian and pancreatic (Hooper et al., 2022). In normal development, EDB-FN is thought to regulate the growth of connective tissue cells (Fukuda et al., 2002). In cancers, EDB-FN can regulate adhesion, angiogenesis and immune cells (Zhou et al., 2025). While EDB-FN is secreted by cancer cells it adheres to their surface enabling its use as a CAR target (Wagner et al., 2021). CAR-T cells targeting EDB-FN had antitumor activity *in vitro* and in xenograft mice (Wagner et al., 2021; Tang et al., 2023).

### Glypican-1 (GPC1) and Glypican-2 (GPC2)

GPC1 and GPC2 are heparansulfate proteoglycans highly expressed in embryogenesis with low expression in many adult tissues (Cao et al., 2024; Chen et al., 2022). GPC1 is upregulated in multiple cancers including lung and prostate (Cao et al., 2024). GPC2 is upregulated in several pediatric cancers including medulloblastoma and adult tumors including brain and lung (Okada et al., 2025; Chen et al., 2022). GPC1 and GPC2 have roles in neuronal development (Jen et al., 2009; Ouchida et al., 2023). In malignancy, GPC1 and GPC2 have roles in cell survival and proliferation (Wang et al., 2019; Chen et al., 2022). GPC1 CAR-T cells combined with PD-1 blockade showed efficacy against colon cancer *in vitro* and *in vivo* (Kato et al., 2020). GPC2 CAR-T cells have anti-tumor activity against *in vitro* and *in vivo* models of neuroblastoma and medulloblastoma (Heitzeneder et al., 2022; Okada et al., 2025).

### Receptor tyrosine kinase-like orphan receptor 2 (ROR2)

Like ROR1, ROR2 is expressed in multiple cancers such as kidney cancer with absent expression in most adult tissues (Weber et al., 2025). During embryogenesis, ROR2 is important in the formation of the limb and nervous system (Al-Shawi et al., 2001).

ROR2 can act as an oncogene by mediating Wnt signaling (Debebe and Rathmell, 2015).

ROR2 CAR-T cells are effective against kidney cancer *in vitro* and *in vivo* (Weber et al., 2025).

While clinical validation is necessary, these emerging targets show great promise. Many of these emerging targets show similarities with those already tested in clinical trials but perhaps with better tumor restriction, perhaps limiting on-target toxicities. EDC-TNC and EDB-FN are unique in being secreted proteins that bind to cancer cells and may potentially reduce antigen heterogeneity; whether this improves CAR-T killing requires further investigation.

## Targeting oncofetal antigens: challenges and strategies

Recent trials of oncofetal-targeting CAR-T cells in solid tumors have been generally well tolerated and have shown encouraging results in limited cancers, particularly neuroblastoma and diffuse midline glioma. While indicating the potential of targeting oncofetal antigens, in many cancers clinical efficacy remains disappointing. To overcome the barriers underlying these limited responses, numerous strategies are being developed to improve outcomes.

### Normal tissue expression

**FIGURE 1:**
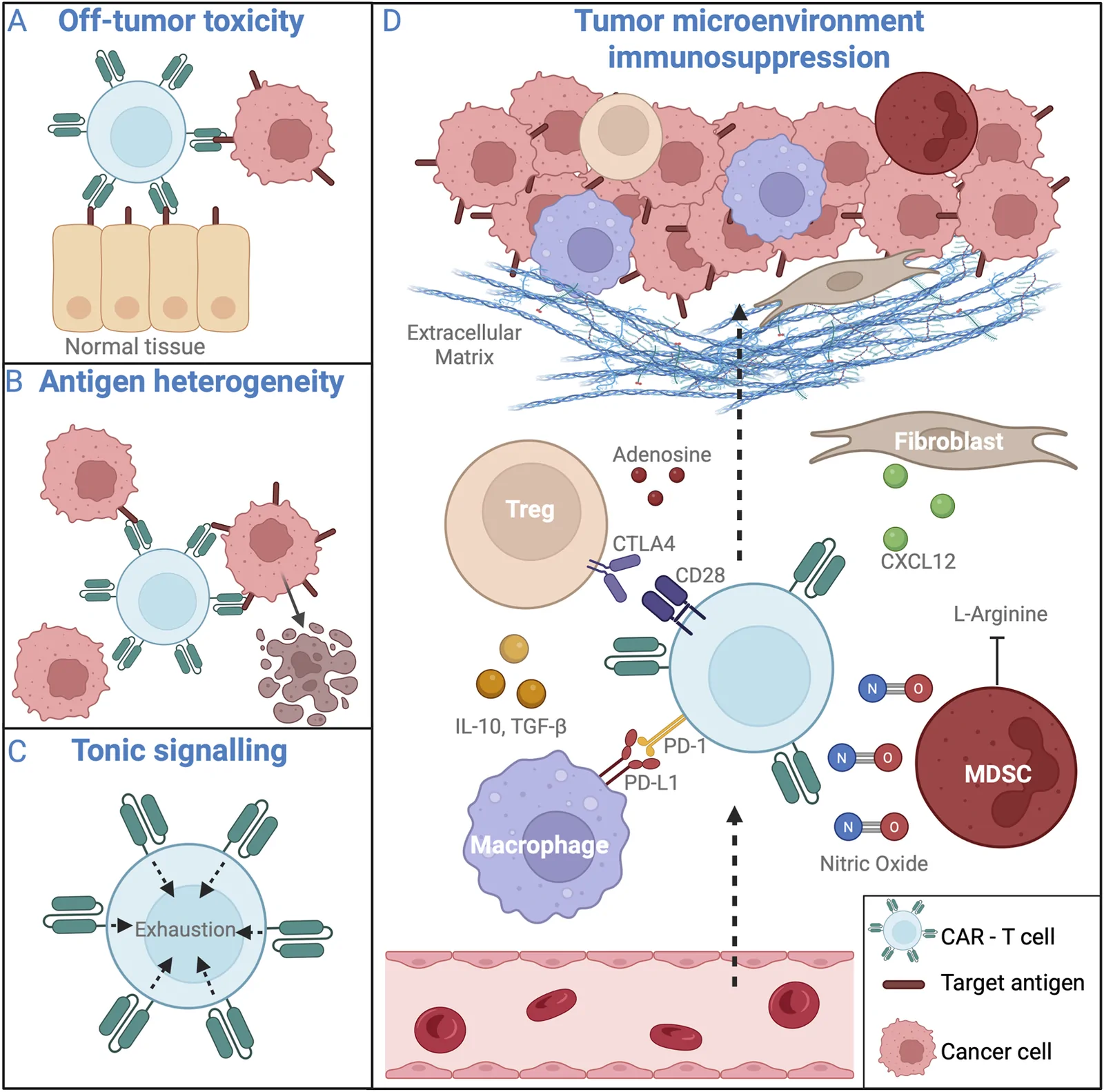
Challenges facing CAR-targeting of oncofetal antigens. **(A)** Normal tissue may express the same oncofetal antigen as cancer cells, often at a lower level. This may lead to chimeric antigen receptor (CAR)-T activation, triggering on-target off-tumor toxicity. **(B)** Antigen heterogeneity may mean that only a subset of cancer cells expresses the target antigen at the necessary level to trigger T cell mediated cytotoxicity. **(C)** Tonic CAR signaling can lead to exhaustion of CAR-T cells before they encounter their target antigen, limiting their ability to kill cancer cells. **(D)** The tumor microenvironment (TME) presents diverse challenges to CAR-T cells. CAR-T cell infiltration into the tumor is often limited by downregulation of endothelial adhesion proteins and the physical barrier created by dense extracellular matrix. Multiple cell types including macrophages, myeloid derived suppressor cells (MDSCs), regulatory T cells (Treg) and fibroblasts are recruited by cancer cells and can inhibit CAR-T cells through multiple mechanisms. Immune checkpoints, e.g., cytotoxic T-lymphocyte associated protein 4 (CTLA4):CD28 and programmed death-ligand 1 (PD-L1): programmed death 1 (PD-1) can inhibit CAR-T activation. Multiple chemokine and cytokines, e.g., transforming growth factor-β (TGF-β) can also suppress CAR-T activation and prevent their migration. Small molecules, e.g., adenosine and nitric oxide are also immunosuppressive. T cell critical nutrients, e.g., L-arginine can also be depleted by MDSCs. Created in BioRender. Smith, S. (2026) https://BioRender.com/vlw4v0l.

Oncofetal antigens were initially discovered due to their presence in fetal and tumor tissues and absence in normal tissues. With the use of modern, more sensitive technologies, it was shown that many oncofetal antigens do have low level expression in normal adult tissues (Southall et al., 1990; Jansen et al., 2024; Zheng et al., 2022; Reinhard et al., 2020). Consequently, on-target off-tumor toxicity is a major concern for oncofetal antigens, and likely responsible for part of the toxicity seen in clinical trials (Figure 1A). Selecting oncofetal antigens with tumor-restricted expression is one method to prevent on-target off-tumor toxicity. Large scale transcriptomic databases of fetal, adult and cancerous tissue are key to identifying oncofetal-restricted expression. While large cancer expression datasets exist for many common cancers, for rarer cancers this data is less abundant. Dedicated efforts combining smaller datasets can enable identification of promising oncofetal antigens in rare cancers (Shaw et al., 2024). Another way to address oncofetal antigen expression in normal tissues is via advanced CAR engineering that enables safer targeting. Examples include “logic gating”, a strategy to prevent activation of CAR-T cells in the presence or absence of a specific molecule, which has the potential to limit on-target off-tumor toxicity. Sandberg et al. developed anti-CEA CAR-T cells which also express a novel leukocyte Ig-like receptor 1 (LIR-1)-based receptor preventing CAR-T activation in the presence of HLA-A*02. They demonstrated selective antitumor efficacy *in vitro* and *in vivo* against cancer cells that do not express HLA-A*02 (Sandberg et al., 2022).

### Tumor-antigen heterogeneity and escape

Few, if any, proteins show universally high expression within every cell of a solid tumor, and this applies to oncofetal antigens (Figure 1B). In addition to limiting efficacy against the tumor, heterogenous expression provides a substrate for tumor evolution in response to selective pressure against the target protein and thus antigen escape (Lin et al., 2024). One strategy to overcome low antigen expression is to target multiple antigens at once. CAR-T cells expressing two CARs targeting TAG72 and CD47 were found to have improved *in vitro* killing of TAG72-low cancer cells (Shu et al., 2021). CD47 is a macrophage “do not eat me signal” expressed on cancer cells but also normal cells. The CD47 CAR in this study was devoid of signaling domains meaning this CAR only enhanced binding to facilitate CAR-T activation through the TAG72 CAR (Shu et al., 2021). There are several other strategies to overcome antigen escape including upregulation of the target antigen and promoting bystander killing (Lin et al., 2025; Lin et al., 2024).

### CAR-T properties

Independent of tumor cells and the TME, CAR-T intrinsic features can affect their persistence, exhaustion and cytotoxic ability. To provide robust tumor control, CAR-T cells need to survive and persist long-term. CAR-T persistence in the blood has been associated with long-term disease-free survival in hematological cancers and neuroblastoma (Shiqi et al., 2023; Li C.-H. et al., 2025). Ensuring consistent antigen exposure is one strategy to enhance persistence but does risk exhaustion. Reinhard et al. combined CLDN6 CAR-T cells with a CLDN6-encoding RNA vaccine and demonstrated in mice that this strategy improved CAR-T expansion, persistence and tumor control (Reinhard et al., 2020). This strategy was utilized in a subsequent clinical trial which did show objective responses, but whether the RNA vaccine improves efficacy over CLDN6 CAR-T cells alone is unclear (Mackensen et al., 2023).

To effectively eliminate cancer cells over prolonged periods, CAR-T cells need to avoid exhaustion. Importantly, the design of CAR-T cells can lead to exhaustion before they encounter the tumor (Figure 1C). For example, tonic CAR signaling can induce early CAR-T exhaustion and limit anti-tumor function (Long et al., 2015). Conditional CAR expression can avoid this tonic signaling. Controlling CEA-CAR expression through hypoxia-responsive elements reduced evidence of exhaustion and improved anti-tumor control in mouse models (Gao et al., 2026). In a phase 1 trial, these hypoxia-responsive CEA-CAR-T cells were administered intra-peritoneally (IP) or intravenously (IV). The dose-limiting toxicity was diarrhea, but even at the doses tested there was an ORR of 23.5% with IP administration, and only 8% with IV (Gao et al., 2026).

CAR-T manufacturing can also affect its performance. There is substantial evidence in hematological malignancies that the starting T cells and the ultimate phenotype of CAR-T cells in the pre-infusion product affects clinical outcome (Fraietta et al., 2018; Locke et al., 2020). The ultimate CAR-T product is a consequence of the manufacturing process; activation and cultivation conditions, duration and cryopreservation all impact the CAR-T product and optimization of these factors may improve CAR-T efficacy (Agliardi et al., 2025).

The intended outcome of CAR-T activation is killing of the target cell. CAR-T cells typically induce apoptosis upon release of granzyme and perforin (Benmebarek et al., 2019). Induction of immunogenic cell death such as necroptosis, pyroptosis or ferroptosis may enable tumor cell killing when they are resistant to apoptosis and trigger the endogenous immune system to enact bystander killing (Humer et al., 2026). For example, the combination of ROR1 CAR-T cells and pharmacological induction of ferroptosis improves tumor control *in vitro* and *in vivo* (Li D. et al., 2025b).

### Optimal CAR - expressing cell

The clinical evidence discussed in this review is dominated by CAR-T cells; whether this is the optimal cell type is unknown. There has been pre-clinical development of oncofetal antigen-targeting CARs expressed on alternative cell types, primarily NK cells and macrophages. GPC3 targeting CAR-macrophages and CAR-NK cells have shown anti-tumor efficacy against HCC in multiple models (Cao et al., 2025; Guan et al., 2024) and CLDN6 targeted CAR-NK cells demonstrated killing of ovarian cancer cells *in vitro* and *in vivo* (Li et al., 2024). There has not been direct comparison between CAR-macrophages, CAR-NK cells and CAR-T cells and it may be that the best cell type for use is dependent on the context, e.g., stage, cancer type, timepoint and prior therapies.

### Tumor microenvironment (TME)

The solid TME is a major obstacle to all CAR-T cells (Figure 1D). Challenges presented by the TME include impaired trafficking, immunosuppressive signals and immune checkpoints (Yang et al., 2025). As a result of several, complex immunosuppressive processes, T cells and other immune cells commonly display dysfunctional phenotypes, preventing effective anti-tumor effector functions. To overcome TME immunosuppression, several strategies are being investigated; one is to “armor” the CAR-T cell, for example, by engineering release of or utilizing signaling driven by cytokines (Hawkins et al., 2021). Many different cytokines have been tested, including IL-15 and IL-18, which were shown to improve anti-tumor efficacy (Hawkins et al., 2021). For example, the efficacy of EDC-TNC targeted CAR-T cells in an osteosarcoma mouse model was significantly enhanced via co-expression of a modular leucine zipper based chimeric cytokine receptor designed to activate IL-18 signaling pathways (Wickman et al., 2024). Furthermore, IL-15 armored anti-GPC3 CAR-T cells have been tested in early phase clinical trials, with the armored construct achieving an ORR of 33% compared to 0% without armoring (Steffin et al., 2025). Concurrent expression of specific transcription factors may also help overcome the obstacles posed by the TME. For example, Runt-related transcription factor 3 (RUNX3) regulates T cell infiltration in the TME, and its co-expression in GPC3 targeting CAR-T cells improves their infiltration and anti-tumor efficacy *in vivo* (Wang et al., 2023). A phase 1 trial of these CAR-T cells did not show unexpected adverse events, but had limited efficacy, with an ORR of 16.7% (Fu et al., 2023). Combination with existing therapies, such as radiotherapy, also provides promise in modulating the TME to improve CAR-T efficacy (Qin et al., 2022). Hepatic artery infusions of CEA CAR-T cells combined with selective internal radiation in a phase 1 trial of patients with heavily pretreated CEA + liver metastases led to median overall survival of 8 months (Katz et al., 2020).

### Patient selection

In addition to the challenges above, choosing the right patients to receive CAR-T cell therapy is critical to its success. Expression of the CAR target on tumor tissue via IHC was a key inclusion factor in most of the clinical trials discussed in this review. However, antigen expression using IHC does not necessarily predict response to CAR-T (Spiegel et al., 2021). Key reasons include spatial and temporal heterogeneity, reliance on single often historical biopsy samples and difficulties defining antigen positivity and minimum antigen density thresholds. These issues have led to the removal of antigen expression as an eligibility criterion in some clinical trials, such as a phase I study investigating mesothelin CAR-T cells (NCT03054298). Novel techniques including quantitative antigen density analyses and imaging-based companion diagnostics may better guide patient selection (Spiegel et al., 2021; Liao et al., 2023).

## Future prospects

Oncofetal antigens are promising targets for CAR-T cell therapies across a wide range of malignancies, including rare tumors. Rare cancers such as sarcomas remain under-researched and have poor outcomes in the metastatic setting. Notably, many oncofetal antigens are expressed across multiple sarcoma subtypes. For example, 5T4 is present in synovial sarcomas, fibrosarcomas, leiomyosarcomas, rhabdomyosarcomas, undifferentiated pleomorphic sarcomas, dermatofibrosarcoma protuberans, and gastrointestinal stromal tumors (Groothuis et al., 2021). Inclusion of patients with rare tumors in clinical trials of oncofetal-targeted therapies may therefore benefit an underserved patient population.

There are multiple obstacles preventing the success of CARs targeting oncofetal antigens, as demonstrated by the disappointing clinical efficacy of 1^st^ and 2^nd^ generation CAR-T cells alone. The strategies discussed above show promise in improving clinical effectiveness. However, one of the major challenges of targeting oncofetal antigens relates to the cancer cells themselves; oncofetal reprogramming is typically not fixed and only a proportion of cancer cells may be in an oncofetal state at any time (Nguyen et al., 2026). Selective pressure against an oncofetal antigen is therefore likely to select for non-fetal-like cell states with low expression of the target antigen. Nevertheless, targeting the treatment-resistant oncofetal cell state with CAR-T cells, either alone or alongside therapies directed against other tumor cell states, may improve tumor control. The plasticity of oncofetal reprogramming also presents an opportunity to increase the proportion of tumor cells expressing target antigens before CAR-T cell therapy. For example, CLDN6 expression in ovarian cancer is influenced by cell density, carboplatin exposure and TGF-β signaling (Kimura et al., 2026). A better understanding of oncofetal reprogramming may therefore enhance the efficacy of oncofetal-targeted CAR-T cells.

## Conclusion

Oncofetal antigens represent a promising class of CAR-T cell targets across both common and rare malignancies. Several have entered clinical evaluation and, although early responses were limited, advances in CAR engineering and combination approaches are beginning to improve therapeutic efficacy. Multiple emerging oncofetal antigens are expanding the target repertoire, and their integration with strategies designed to overcome key barriers to CAR-T cell activity holds considerable promise. A deeper understanding of oncofetal reprogramming may further reveal novel targets and therapeutic opportunities.
